# Prevalence of Hypoxic Ischemic Encephalopathy and Multiorgan Dysfunction in Late Preterm and Term Infants Receiving Resuscitation Beyond Initial Steps at Birth

**DOI:** 10.7759/cureus.103810

**Published:** 2026-02-17

**Authors:** Parul Sohane, Shakal N Singh, Himanshu Gupta, Manisha Verma, Shalini Tripathi, Arpita Bhriguvanshi

**Affiliations:** 1 Department of Paediatrics, King George's Medical University, Lucknow, IND

**Keywords:** central nervous system, hypoxic ischemic encephalopathy, initial steps, late preterm infants, multiorgan dysfunction, perinatal asphyxia, resuscitation, term infants

## Abstract

Background and objective: Perinatal asphyxia is a leading cause of neonatal morbidity and mortality, frequently resulting in hypoxic-ischemic encephalopathy (HIE) and multiorgan dysfunction (MOD). During asphyxia, the “diving reflex” preferentially redistributes blood flow to vital organs, predisposing other organ systems to ischemic injury. Data on the burden and pattern of MOD among resuscitated late preterm and term neonates remain limited. The primary objective of this study was to determine the frequency and severity of HIE in late preterm and term neonates requiring resuscitation beyond the initial steps at birth. The secondary objectives were to evaluate the prevalence and pattern of MOD in these infants and to assess the association between HIE severity, organ dysfunction, and mortality.

Materials and methods: A prospective observational study was conducted over one year in the neonatal unit and neonatal intensive care unit (NICU) of a tertiary-care hospital, King George’s Medical University (KGMU), Lucknow, India. A total of 164 neonates (≥34 weeks’ gestation) who required resuscitation beyond initial steps were enrolled after obtaining informed parental consent. Demographic, perinatal, and clinical data were recorded. Organ dysfunction was evaluated using clinical and biochemical criteria for the central nervous, cardiovascular, renal, hepatic, respiratory, hematologic, and metabolic systems. The severity of HIE was graded using Sarnat and Sarnat staging. Statistical analysis was performed using IBM SPSS Statistics software, version 26 (IBM Corp., Armonk, NY, USA), with p <0.05 considered significant.

Results: Among 164 neonates requiring resuscitation, 40% (66/164) developed HIE, with Stage III being most common (51.5%). MOD was frequent, with metabolic derangements (81.1%) and renal (55.5%) being the most prevalent. Overall mortality was 20.7% (34/164), highest among neonates with cardiovascular dysfunction (54.9%) and HIE III (79.4%). The intensity of resuscitation correlated with organ involvement: prolonged positive pressure ventilation (PPV) >1 min, intubation, chest compressions, and drug use were significantly associated with higher rates of central nervous system (CNS), cardiovascular, renal, respiratory, hematological, gastrointestinal, and metabolic dysfunction (p<0.05).

Conclusion: HIE and MOD are common in late preterm and term neonates requiring resuscitation beyond initial steps, with the severity of HIE closely linked to the extent of organ involvement and mortality. Early recognition and close monitoring of MOD are essential to improve outcomes in this high-risk population.

## Introduction

Perinatal asphyxia remains a major global health problem and a leading contributor to neonatal morbidity and mortality. It accounts for approximately 9% of under-five deaths worldwide and is among the top three causes of neonatal mortality, alongside prematurity and infections [1]. Globally, hypoxia is responsible for nearly 1.2 million intrapartum stillbirths, and approximately 9.5% of liveborn infants require some form of resuscitation at birth [2]. National data from the National Neonatal Perinatal Database (NNPD) indicate that about 1.4% of neonates develop clinical hypoxic-ischemic encephalopathy (HIE), with perinatal asphyxia contributing to nearly 28.8% of neonatal deaths [3]. Notably, the majority of asphyxia-related deaths occur early, with approximately 98% occurring within the first week of life and nearly three-quarters within the first 24 hours [4].

Beyond its immediate mortality burden, perinatal asphyxia is a major cause of long-term neurological morbidity, including cerebral palsy, cognitive impairment, and other neuromotor disabilities [5]. Severe or prolonged hypoxic-ischemic insults before or during birth can lead to HIE, a clinical syndrome characterized by impaired neurological function in the early neonatal period in term or near-term infants [6]. Importantly, the pathophysiology of HIE is not confined to the central nervous system (CNS); systemic hypoxia and ischemia result in widespread cellular injury, predisposing multiple organ systems to dysfunction.

The compensatory response to acute hypoxia, known as the “diving reflex,” redirects blood flow from non-vital organs (such as the kidneys, liver, and intestines) to vital organs (the brain, heart, and adrenal glands) [7]. While this mechanism temporarily preserves oxygen delivery to critical tissues, it also predisposes other organs to ischemic damage, leading to multiorgan dysfunction (MOD). The spectrum of systemic injury may include renal tubular necrosis, hepatic enzyme elevation, myocardial ischemia, pulmonary hypertension, adrenal hemorrhage, and coagulopathy [8].

Despite extensive research on HIE, discrepancies persist regarding diagnostic criteria, inclusion standards, and the classification of organ dysfunction across studies [9]. Consequently, the true prevalence and impact of MOD in asphyxiated neonates remain uncertain. Understanding these relationships is vital, as the presence and extent of MOD are important prognostic indicators for both mortality and long-term neurodevelopmental outcomes [10].

The present study was undertaken to determine the frequency and severity of HIE among late preterm and term neonates requiring resuscitation beyond the initial steps at birth and to evaluate the pattern and prevalence of MOD in these infants. Additionally, it aims to explore the association between HIE severity, organ dysfunction, and mortality, thereby providing insights that may inform neonatal resuscitation and management strategies.

## Materials and methods

This prospective observational study was conducted over a one-year period in the neonatal unit and neonatal intensive care unit (NICU) of King George’s Medical University (KGMU), a tertiary-care teaching hospital in Lucknow, India. The study population comprised neonates admitted to the neonatal unit or NICU who required resuscitation beyond the initial steps at birth. Neonates with a gestational age of 34 weeks or more who required advanced resuscitative interventions and whose parents or legal guardians provided written informed consent were eligible for inclusion. Neonates with major congenital anomalies or those for whom informed consent could not be obtained were excluded. Ethical approval for the study was granted by the Institutional Ethics Committee of KGMU (Ref. No. 107th ECM II B Thesis/P30 dated 31/05/21; No. 522/Ethics/2021). Written informed consent was obtained from parents or guardians in their vernacular language prior to enrollment.

Perinatal asphyxia was diagnosed when two or more predefined criteria were fulfilled, including abnormal fetal heart rate patterns during the intrapartum period, evidence of metabolic acidosis on cord blood or neonatal arterial blood gas analysis within one hour of birth with a pH below 7.0 or a base deficit greater than 12 mEq/L, and the requirement for resuscitation beyond the initial steps at birth [11].

Neonatal encephalopathy was defined as a clinically recognizable syndrome of disturbed neurological function manifesting within the first few days of life in neonates born at or beyond 35 weeks of gestation. This condition was characterized by abnormalities in consciousness, tone, reflexes, respiratory function, or the presence of seizures [11]. HIE was considered a specific subtype of neonatal encephalopathy resulting from systemic hypoxemia and/or impaired cerebral perfusion secondary to perinatal or intrapartum hypoxia-ischemia. The severity of HIE was classified using the Sarnat and Sarnat staging system [12, 13].

Study procedures and resuscitation protocol

All deliveries were attended by a trained neonatal resuscitation team consisting of a pediatric resident and a nursing assistant, in accordance with the Indian Neonatal Resuscitation Programme guidelines based on International Liaison Committee on Resuscitation recommendations [14].

Initial steps of neonatal resuscitation included providing warmth by placing the baby under a radiant heat source, positioning the head in a “sniffing” position to open the airway, clearing the airway, if necessary, with a bulb syringe or suction catheter, drying the baby, and tactile stimulation.

Resuscitation beyond initial steps included oxygen supplementation or continuous positive airway pressure, positive pressure ventilation, endotracheal intubation, chest compressions, and administration of intravenous fluids or medications as indicated. Detailed information regarding gestational age, birth weight, sex, Apgar scores, and the specific resuscitative interventions performed was systematically recorded for each neonate.

Clinical monitoring and data collection

Comprehensive maternal, antenatal, and intrapartum histories were obtained, including maternal age, parity, antenatal complications such as anemia, hypertension, diabetes, and infections, as well as intrapartum factors such as meconium-stained amniotic fluid, prolonged rupture of membranes, and fetal distress. Neonates were monitored for vital signs, neurological status, and evidence of systemic involvement at birth, at 24 hours of life, at discharge, and again at one-month follow-up.

Assessment of organ dysfunction

All organ systems were evaluated using predefined clinical and biochemical criteria. The severity of dysfunction in each system was graded on a standardized scale ranging from 0 to 3, with increasing scores reflecting greater severity of organ involvement.

Neurological involvement was assessed through detailed clinical examination focusing on tone, level of consciousness, and the presence of seizure activity. The severity of HIE was classified according to the Sarnat and Sarnat staging system [13].

Cardiovascular system (CVS) involvement was evaluated using both clinical and biochemical parameters. Clinical assessment included the presence of tachycardia, dysrhythmias, or the requirement for vasoactive drug support. Biochemical assessment was performed by measuring serum troponin T levels at 24 hours of life using the electrochemiluminescence immunoassay method. Cardiovascular severity was graded as score 0 when serum troponin T levels were below 0.10 µg/L and no vasoactive drugs were required, indicating no cardiovascular involvement. A score of 1 was assigned when serum troponin T levels were between 0.10 and 0.24 µg/L or when a single vasoactive drug was required for less than 24 hours, indicating mild cardiovascular involvement. A score of 2 represented moderate involvement and was assigned when serum troponin T levels were 0.24 µg/L or higher or when a single vasoactive drug was required for 24 hours or more. A score of 3 indicated severe cardiovascular involvement and was assigned when two or more vasoactive drugs were required [15].

Renal system involvement was assessed using clinical evaluation of urine output measured in milliliters per kilogram per hour and biochemical measurement of serum creatinine levels at admission and again at 24 hours using the enzymatic colorimetric method. Renal severity was graded as score 0 when serum creatinine levels were below 1.0 mg/dL and urine output was at least 1.0 mL/kg/hour, indicating normal renal function. A score of 1, representing mild renal dysfunction, was assigned when serum creatinine levels ranged from 1.0 to 1.25 mg/dL with urine output between 0.99 and 0.51 mL/kg/hour. A score of 2 indicated moderate renal dysfunction and was assigned when serum creatinine levels ranged from 1.26 to 1.5 mg/dL with urine output of 0.5 mL/kg/hour or less. Severe renal dysfunction was defined as a score of 3 and was assigned when serum creatinine exceeded 1.5 mg/dL or increased by 0.3 mg/dL or more within 24 hours, in association with oliguria.

Respiratory system involvement was assessed based on the requirement for respiratory support, excluding support required solely due to central apnea or drug effects. A respiratory severity score of 0 indicated that no respiratory support was required. A score of 1 was assigned to neonates who required non-invasive ventilation or an inspired oxygen fraction below 0.4 for less than 24 hours, indicating mild respiratory involvement. A score of 2 represented moderate respiratory involvement and was assigned to neonates who required mechanical ventilation for 24 hours or more or an inspired oxygen fraction of 0.4 or higher. Severe respiratory involvement was defined as a score of 3 and was assigned when high-frequency oscillatory ventilation was required [16].

Gastrointestinal system involvement was evaluated using both clinical and biochemical parameters. Clinical assessment included the presence of abdominal distension, gastrointestinal bleeding, or necrotizing enterocolitis. Biochemical evaluation consisted of measuring serum aspartate aminotransferase and bilirubin levels at birth and at 24 hours using the International Federation of Clinical Chemistry method [17,18]. Gastrointestinal severity was graded as score 0 when serum aspartate aminotransferase levels were below 100 U/L, indicating no involvement. A score of 1 indicated mild involvement and was assigned when serum aspartate aminotransferase levels were 100 U/L or higher. A score of 2 represented moderate involvement and was assigned when serum aspartate aminotransferase levels were 500 U/L or higher. Severe gastrointestinal involvement was defined as a score of 3 and was assigned when serum aspartate aminotransferase levels reached or exceeded 1000 U/L.

Hematologic system involvement was assessed using complete blood counts and coagulation parameters, including prothrombin time, international normalized ratio, and activated partial thromboplastin time, measured at admission and at 24 hours. A hematologic severity score of 0 indicated normal status, defined by a white blood cell count between 4.5 and 30 × 10⁹/L, a platelet count of at least 150 × 10⁹/L, and an activated partial thromboplastin time of 45 seconds or less. A score of 1 was assigned for mild hematologic abnormality and included white blood cell counts below 4.5 or above 30 × 10⁹/L, platelet counts between 51 and 149 × 10⁹/L, or an activated partial thromboplastin time exceeding 45 seconds. A score of 2 represented moderate thrombocytopenia with platelet counts between 21 and 50 × 10⁹/L. Severe hematologic involvement was defined as a score of 3 and was assigned when platelet counts were 20 × 10⁹/L or lower.

Metabolic dysfunction was evaluated using clinical evidence of hypoglycemia and biochemical assessment of blood glucose and serum electrolytes. Hypoglycemia was defined as a random blood sugar level <40 mg/dL. Serum sodium, potassium, and calcium levels were measured at admission and at 24 hours of life using the indirect ion-selective electrode method in the Department of Pathology.

Each metabolic parameter was graded on a standardized three-point scale (0-2) based on predefined neonatal reference thresholds. A grade of 0 was assigned when values were within normal limits. Grade 1 represented minor electrolyte derangement, and grade 2 represented major electrolyte derangement.

For serum sodium, values of 135-145 mmol/L were graded as 0; mild hypernatremia (146-159 mmol/L) or mild hyponatremia (134-121 mmol/L) were graded as 1; and severe hypernatremia (≥160 mmol/L) or severe hyponatremia (≤120 mmol/L) were graded as 2.

For serum potassium, values of 3.5-5.5 mmol/L were graded as 0; mild hyperkalemia (5.6-6.4 mmol/L) or mild hypokalemia (3.4-2.6 mmol/L) were graded as 1; and severe hyperkalemia (≥6.5 mmol/L) or severe hypokalemia (≤2.5 mmol/L) were graded as 2.

For serum calcium, values of 4.0-5.2 mmol/L were graded as 0; mild hypercalcemia (5.25-5.97 mmol/L) or mild hypocalcemia (3.96-2.84 mmol/L) were graded as 1; and severe hypercalcemia (≥6.0 mmol/L) or severe hypocalcemia (≤2.8 mmol/L) were graded as 2.

Increasing grades reflected increasing severity of metabolic derangement and were used to assess the extent of metabolic involvement in neonates with hypoxic-ischemic encephalopathy.

Laboratory investigations

All laboratory investigations were performed in the Departments of Biochemistry and Pathology using standardized, routinely calibrated automated analyzers. Hematological parameters, serum cardiac troponin T, renal and hepatic function tests, coagulation profiles, and inflammatory markers were assessed using validated laboratory protocols in accordance with institutional standards. Blood cultures were processed using automated culture systems for the detection of aerobic bacterial pathogens.

Outcome measures

The primary outcomes included the occurrence and severity of HIE and the incidence and pattern of MOD. Secondary outcomes included the association between HIE severity, MOD, and mortality, as well as short-term outcomes defined as survival or death prior to hospital discharge.

Sample size and statistical analysis

The sample size was calculated assuming a 50% prevalence of MOD among asphyxiated neonates, with an absolute precision of 7.5% and a 95% confidence interval, yielding a minimum required sample size of 164 neonates, calculated using n-MASTER version 2.0 (CMC Vellore, Vellore, India). Data were entered into Microsoft Excel (Microsoft Corp., Redmond, WA, USA) and analyzed using IBM SPSS Statistics software, version 26.0 (IBM Corp., Armonk, NY, USA). Continuous variables were expressed as mean with standard deviation or median with interquartile range, depending on data distribution, while categorical variables were presented as percentages. Appropriate statistical tests, including the chi-square test, Fisher’s exact test, independent t-test, or Mann-Whitney U test, were applied as indicated. A p-value less than 0.05 was considered statistically significant.

## Results

A total of 164 neonates requiring resuscitation beyond the initial steps at birth were enrolled based on predefined inclusion and exclusion criteria. All enrolled neonates were followed up to assess the occurrence and severity of HIE, as well as to evaluate MOD based on standardized clinical and biochemical parameters. The final outcomes were recorded as discharge, death, or loss to follow-up.

The overall prevalence of perinatal asphyxia (based on the adopted definition) was 71% (118/164). Among the study population, 40% (66/164) of neonates developed HIE, while 59.7% (98/164) had no features of HIE. The mean maternal age was comparable between the two groups (26.18 ± 3.56 vs. 26.59 ± 3.90 years, p = 0.495), and vaginal delivery was the predominant mode in both (62.1% vs. 62.2%, p = 0.987). However, cesarean delivery for fetal distress was significantly more frequent in the HIE group (75.6% vs. 46.3%, p = 0.017), indicating a strong intrapartum hypoxia component. Regular antenatal check-ups were significantly lower among mothers of HIE neonates (36.4% vs. 57.1%, p = 0.009), suggesting poor antenatal surveillance as a risk factor. Meconium-stained liquor was also more common in the HIE group (60% vs. 22.2%). Neonates with HIE had a significantly lower five-minute Apgar score (4.95 ± 1.11 vs. 6.29 ± 1.06, p < 0.0001) and a higher incidence of abnormal fetal heart rate tracings (p = 0.0068). Gestational age and birth weight were comparable between groups (Table 1).

**Table 1:** Clinicodemographic maternal and neonatal characteristics in HIE and no-HIE groups Data are expressed as mean ± standard deviation (SD) or number (percentage). HIE: hypoxic-ischemic encephalopathy; LSCS: lower segment cesarean section; APH: antepartum hemorrhage; FHR: fetal heart rate *Significant at p < 0.05.

Parameter	HIE (n = 66)	No-HIE (n = 98)	Test Statistic	p-Value
Maternal Parameters				
Maternal age (years)	26.18 ± 3.56	26.59 ± 3.90	t = 0.6835	0.4953
Mode of delivery				
Vaginal	41 (62.12%)	61 (62.24%)	χ² = 0.00026	0.9872
Cesarean section	25 (37.88%)	37 (37.76%)		
Indication for LSCS				
Breech	3 (7.32%)	11 (26.83%)	χ² = 17.05	0.0171*
Eclampsia	1 (2.44%)	0 (0%)		
Fetal distress	31 (75.61%)	19 (46.34%)		
Fetal heart disease	1 (2.44%)	0 (0%)		
Nuchal cord	1 (2.44%)	0 (0%)		
Low general condition of the mother	0 (0%)	4 (9.76%)		
Previous LSCS	3 (7.32%)	7 (17.07%)		
Prolonged labour	1 (1.52%)	0 (0%)		
Booking status				
Regular antenatal check-up	24 (36.36%)	56 (57.14%)	χ² = 6.816	0.0090*
No regular antenatal check-up	42 (63.64%)	42 (42.86%)		
Obstetric Conditions				
APH	0 (0%)	2 (11.11%)	χ² = 8.866	0.1145
Pregnancy-induced hypertension	1 (6.67%)	3 (3.06%)		
Meconium-stained liquor	9 (60.00%)	4 (22.22%)		
Clinical chorioamnionitis	5 (33.33%)	9 (50.00%)		
Neonatal Parameters				
Gestational age (weeks)	37.23 ± 1.99	36.75 ± 1.98	t = 1.519	0.1306
Apgar score at 5 minutes	4.95 ± 1.11	6.29 ± 1.06	t = 7.789	<0.0001*
Birth weight (g)	3158.83 ± 404.77	2378.46 ± 545.53	t = 1.885	0.0612
FHR pattern				
Normal	9 (13.64%)	70 (71.43%)	χ² = 12.19	0.0068*
Fetal distress	1 (1.52%)	2 (2.04%)		
Pathological	8 (12.12%)	26 (26.53%)		
Suspicious	18 (27.27%)	30 (30.61%)		

**Figure 1:**
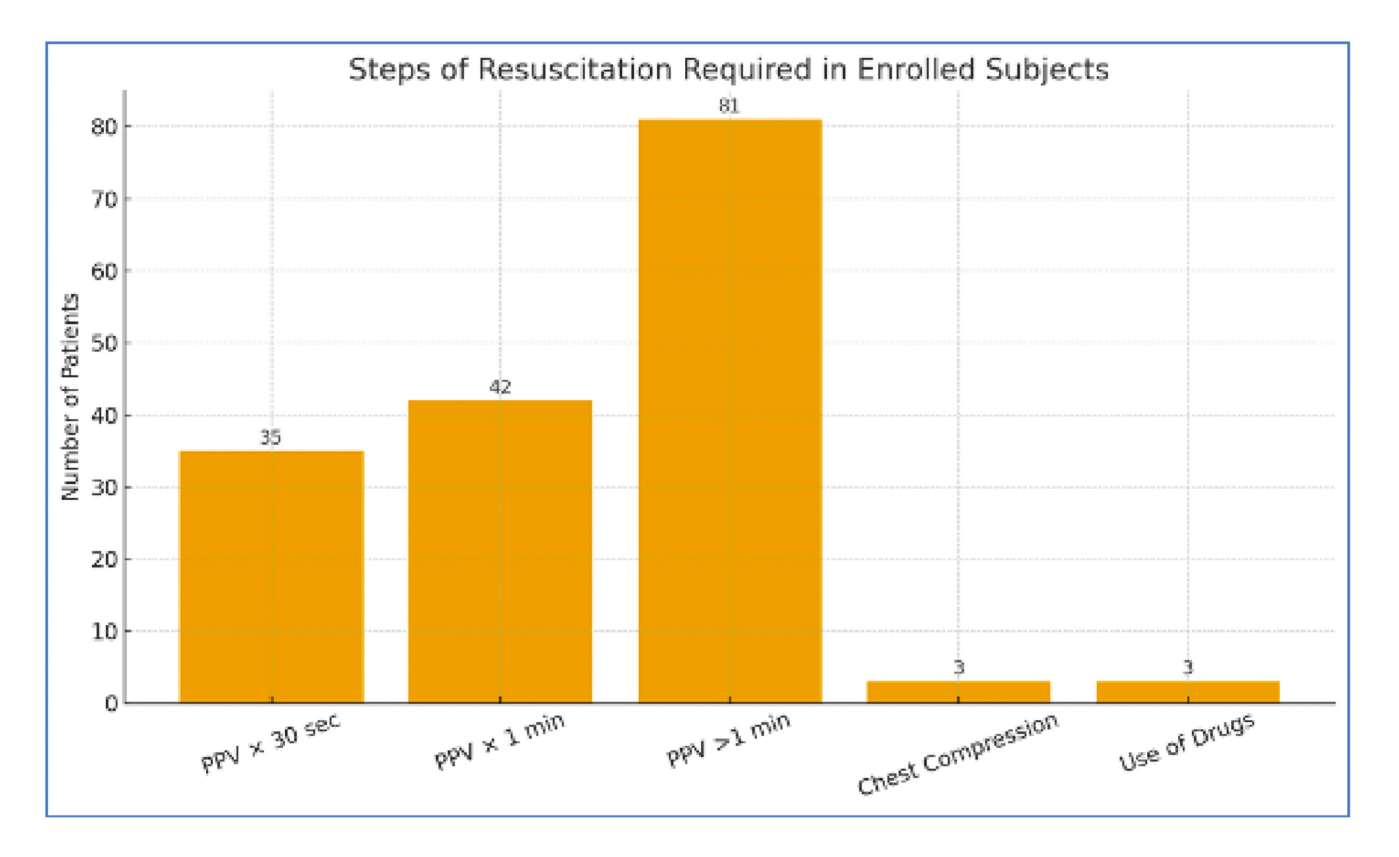
Steps of resuscitation required in enrolled subjects PPV:  positive pressure ventilation. PPV × 30 sec: PPV provided for 30 seconds; PPV × 1 min: PPV provided for one minute; PPV >1 min: PPV required for more than one minute

Among 164 enrolled neonates requiring resuscitation beyond initial steps, 21.3% (n=35) required positive pressure ventilation (PPV) for ≤30 seconds, 25.6% (n=42) for about one minute, and 49.3% (n=81) required PPV >1 minute with intubation. Only 1.8% (n=3) needed chest compressions, and all three received adrenaline (Figure 1).

Most neonates required two cycles of bag-mask ventilation (89, 54.3%), and 53.1% required endotracheal intubation. The majority did not require chest compressions (158, 96.3%) or pharmacological intervention (161, 98.2%). A statistically significant difference was observed in the need for chest compressions and drug administration among the groups.

Among 66 neonates with HIE, staging by the Sarnat and Sarnat classification showed 21.2% (n=14) with Stage I (mild), 27.2% (n=18) with Stage II (moderate), and 51.5% (n=34) with Stage III (severe) encephalopathy. In Stage I, five neonates received BMV (≤1 min), seven required intubation with PPV >1 min, one received chest compressions, and one required drugs. Among Stage II neonates, five received BMV, 12 required intubation with PPV >1 min, and one required chest compressions; none received drugs. All Stage III neonates required intubation with PPV >1 min, with one needing chest compressions and two receiving drugs. Resuscitation intensity differed significantly across HIE stages.

A total of 164 neonates requiring resuscitation were enrolled, and organ dysfunction was assessed using predefined clinical and biochemical parameters (Table 2). Metabolic derangements were most common (81.1%, n=133), followed by renal (55.5%, n=91), respiratory (51.2%, n=84), hematological (42.7%, n=70), CNS (40.2%, n=66), gastrointestinal (32.9%, n=54), and cardiovascular dysfunction (31.1%, n=51). The severity of organ dysfunction differed across HIE stages, with most neonates exhibiting multiorgan involvement in the HIE III subgroup.

**Table 2:** Frequency and severity of multiorgan dysfunction among resuscitated neonates (N = 164) Data expressed as number (percentage). CNS: central nervous system; HIE: hypoxic-ischemic encephalopathy; CVS: cardiovascular system; DIC: disseminated intravascular coagulation; GI: gastrointestinal; NEC: necrotizing enterocolitis; SGOT: serum glutamic oxaloacetic transaminase; MV: mechanical ventilation; FiO₂: fraction of inspired oxygen; h: hours

Organ System	Overall n (%)	Key Parameter	Value n (%)	Severity Pattern
CNS	66 (40.2%)	HIE Stage I	14 (8.5%)	Predominantly HIE III
		HIE Stage II	18 (11.0%)	
		HIE Stage III	34 (20.7%)	
CVS	51 (31.1%)	Troponin-T <0.1 µg/L	139 (84.8%)	Mostly HIE III
		Troponin-T 0.1–0.24 µg/L	20 (12.2%)	
		Troponin-T >0.24 µg/L	7 (4.3%)	
		No vasoactive drug	126 (76.8%)	
		One drug <24 h	11 (6.7%)	
		One drug >24 h	12 (7.3%)	
		≥2 vasoactive drugs	15 (9.1%)	
		Arrhythmia present	9 (5.5%)	
Renal	91 (55.5%)	Urine output ≥1 mL/kg/h	142 (86.6%)	Mostly HIE III
		Urine output 0.51–0.99 mL/kg/h	17 (10.4%)	
		Urine output ≤0.5 mL/kg/h	5 (3.0%)	
		Serum creatinine <1 mg/dL	79 (48.2%)	
		Serum creatinine 1–1.25 mg/dL	34 (20.7%)	
		Serum creatinine 1.26–1.5 mg/dL	18 (11.0%)	
		Serum creatinine >1.5 mg/dL or ↑0.3/24 h	33 (20.1%)	
Hematological	70 (42.7%)	Disseminated intravascular coagulation	7 (4.3%)	Higher in HIE III
		Normal leukocyte count	154 (93.9%)	
		Normal platelet count	125 (76.2%)	
		Normal activated partial thromboplastin time	117 (71.3%)	
GI	54 (32.9%)	Abdominal distension	3 (1.8%)	Mostly HIE III
		Gastrointestinal bleed	9 (5.5%)	
		Necrotizing enterocolitis	0 (0%)	
		SGOT <100 U/L	96 (58.5%)	
		SGOT 100–500 U/L	49 (29.8%)	
		SGOT >500 U/L	19 (11.5%)	
Respiratory	84 (51.2%)	No respiratory support	80 (48.8%)	Mostly HIE III
		Non-invasive or short mechanical ventilation	31 (18.9%)	
		Mechanical ventilation ≥24 h or FiO₂ ≥0.4 ≥24 h	53 (32.3%)	
Metabolic	133 (81.1%)	Hypoglycemia	7 (4.3%)	Mostly HIE III
		Sodium – normal	77 (46.9%)	
		Sodium – minor abnormality	78 (47.6%)	
		Sodium – major abnormality	7 (4.3%)	
		Potassium – normal	95 (57.9%)	
		Potassium – minor abnormality	46 (28.1%)	
		Potassium – major abnormality	23 (14.0%)	
		Calcium – normal	72 (43.9%)	
		Calcium – minor abnormality	70 (42.7%)	
		Calcium – major abnormality	22 (13.4%)	

Regarding association with HIE severity, most neonates with CNS, CVS, renal, respiratory, and metabolic dysfunction were in the HIE III subgroup, followed by HIE II and HIE I. Hematological involvement was higher in HIE III, though not statistically significant. GI dysfunction was highest in HIE III, with more involvement in HIE I than in HIE II (Table 2).

Among 164 neonates requiring resuscitation, MOD was common, with metabolic derangements observed in 81.1% (n=133), renal dysfunction in 55.5% (n=91), respiratory in 51.2% (n=84), hematological in 42.7% (n=70), CNS in 40.2% (n=66), gastrointestinal in 32.9% (n=54), and cardiovascular in 31.1% (n=51). Overall mortality was 20.7% (34/164), with the highest proportion among neonates with cardiovascular dysfunction (54.9%, 28/51), followed by gastrointestinal (40.7%, 22/54), hematological (35.7%, 25/70), renal (35.2%, 32/91), respiratory (34.5%, 29/84), and metabolic dysfunction (24.1%, 32/133) (Table 3).

**Figure 2:**
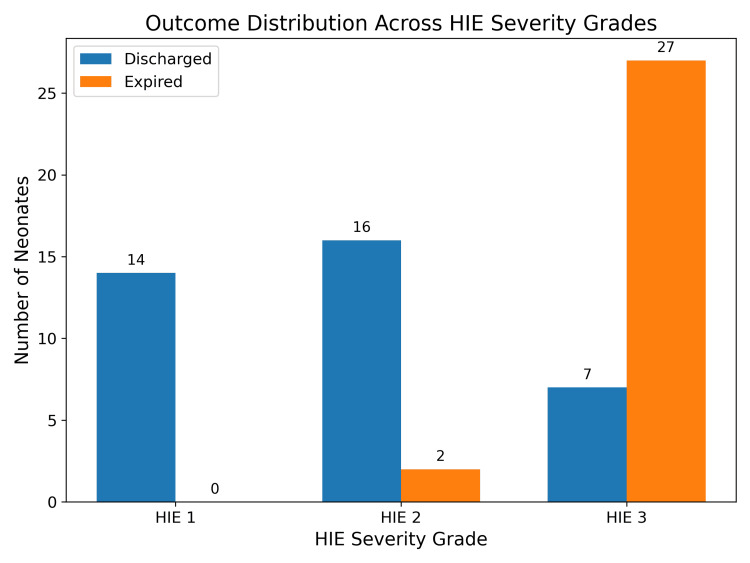
Central nervous system outcome among neonates categorized in hypoxic-ischemic encephalopathy (HIE) subgroups (discharged vs. expired).

CNS dysfunction correlated strongly with HIE severity: all HIE I neonates (n=14, 100%) survived, 11.1% of HIE II neonates (2/18) died, and 79.4% of HIE III neonates (27/34) died (p<0.0001) (Figure 2).

Similarly, multiorgan involvement was more prevalent in HIE III neonates, with 88.2% exhibiting cardiovascular or renal dysfunction and 91.2% showing respiratory involvement. Renal, respiratory, and metabolic dysfunctions showed statistically significant associations with mortality (p<0.05), while hematological and gastrointestinal dysfunctions had moderate associations (Table 3).

**Table 3:** Association of organ dysfunction and HIE stage with mortality in neonates (N = 164). Data expressed as number (percentage). CNS: central nervous system; HIE: hypoxic-ischemic encephalopathy; CVS: cardiovascular system; GI: gastrointestinal The chi-square test was used to assess the association between organ dysfunction or HIE stage and mortality. An asterisk (*) indicates statistical significance at p < 0.05.

Organ System/Parameter	Discharged n (%)	Expired n (%)	Chi-square (χ²)	p-value
CVS (n = 51)	23 (45.09)	28 (54.91)	0.2433	0.4932
Renal (n = 91)	59 (64.83)	32 (35.16)	4.074	0.0435*
Hematological (n = 70)	45 (64.28)	25 (35.71)	3.103	0.0782
GI system (n = 54)	32 (59.25)	22 (40.74)	0.9339	0.3338
Respiratory system (n = 84)	55 (65.47)	29 (34.52)	4.123	0.0423*
Metabolic (n = 133)	101 (75.93)	32 (24.06)	19.13	<0.0001*
CNS HIE stage I (n = 14)	14 (100.00)	0 (0.00)	36.22	<0.0001*
CNS HIE stage II (n = 18)	16 (88.89)	2 (11.11)		
CNS HIE stage III (n = 34)	7 (20.59)	27 (79.41)		

Among 164 neonates requiring resuscitation, the frequency of organ dysfunction increased with the intensity of resuscitation (Table 4). Short-duration PPV (≤30 sec, n=35; ≤1 min, n=42) was associated with lower rates of dysfunction: CNS involvement in 20% and 11.9%, cardiovascular in 5.7% and 11.9%, renal in 28.6% and 35.7%, hematological in 17.1% and 33.3%, gastrointestinal in 5.7% and 16.7%, respiratory in 22.9% and 21.4%, and metabolic in 71.4% and 76.2%, with no statistically significant associations (p>0.05). In contrast, prolonged PPV >1 min with intubation (n=81) was significantly associated with higher dysfunction: CNS 62.96%, cardiovascular 48.15%, renal 76.54%, hematological 56.79%, gastrointestinal 51.85%, respiratory 76.54%, and metabolic 87.65% (all p<0.05). Chest compressions (n=3) and drug administration (n=3) were associated with 100% CNS involvement; chest compressions also showed 100% hematological involvement and 66.7% cardiovascular involvement, while drug use was associated with 100% cardiovascular dysfunction. These findings indicate that escalation of resuscitation measures, including prolonged PPV, chest compressions, and drug use, is strongly associated with higher rates of MOD in neonates.

**Table 4:** Relation of steps of resuscitation with different organ dysfunction (N=164) Data expressed as number (percentage). PPV: positive pressure ventilation; CNS: central nervous system; CVS: cardiovascular system; GI: gastrointestinal system. An asterisk (*) indicates a statistically significant association (p < 0.05) between the respective organ/system involvement and the need for advanced resuscitation interventions (prolonged PPV > 1 minute with intubation, chest compressions, or drug administration), as determined by the chi-square test.

Organ/System	PPV ≤30 sec (n=35)	PPV ≤1 min (n=42)	PPV >1 min + Intubation (n=81)	No PPV >1 min (n=83)	Chest Compression (n=3)	No Chest Compression (n=161)	Drug Use (n=3)	No Drug Use (n=161)
CNS	7 (20%)	5 (11.9%)	51 (62.96%)*	18 (21.69%)	3 (100%)*	66 (40.99%)	3 (100%)*	66 (40.99%)
CVS	2 (5.7%)	5 (11.9%)	39 (48.15%)*	12 (14.46%)	2 (66.7%)	49 (30.43%)	3 (100%)*	48 (29.81%)
Renal	10 (28.57%)	15 (35.71%)	62 (76.54%)*	29 (34.94%)	2 (66.7%)	89 (55.28%)	2 (66.7%)	89 (55.28%)
Hematological	6 (17.14%)	14 (33.33%)	46 (56.79%)*	24 (28.92%)	3 (100%)*	67 (41.61%)	1 (33.33%)	69 (42.86%)
GI system	2 (5.71%)	7 (16.67%)	42 (51.85%)*	12 (14.46%)	1 (33.3%)	53 (32.92%)	1 (33.3%)	53 (32.92%)
Respiratory	8 (22.86%)	9 (21.43%)	62 (76.54%)*	22 (26.51%)	3 (100%)	81 (50.31%)	2 (66.7%)	82 (50.93%)
Metabolic	25 (71.43%)	32 (76.19%)	71 (87.65%)*	62 (74.70%)	2 (66.7%)	131 (81.37%)	2 (66.7%)	131 (81.37%)

## Discussion

This prospective observational study assessed the prevalence and severity of HIE and associated MOD in late preterm and term neonates requiring resuscitation beyond initial steps at birth. A total of 3,400 live births were screened during the study period, of which 164 neonates required resuscitation beyond initial steps and were enrolled. The findings corroborate prior research demonstrating a high burden of MOD in neonates with perinatal asphyxia and HIE. Alsina et al. [15] reported that perinatal hypoxia is a major contributor to MOD, with most neonates affected by HIE developing dysfunction across multiple organ systems. Similarly, consensus-based criteria for diagnosing intrapartum asphyxia include MOD as a major component [19], reflecting the physiological underpinning of the diving reflex, which preserves perfusion to vital organs at the expense of others.

Among the 3,400 live births, perinatal asphyxia was observed in 118 (3.47%) neonates, and HIE in 66 (1.94%). Among neonates requiring resuscitation beyond initial steps, 40.2% developed HIE, with Stage III being the most common (51.5%). These rates are higher than those reported in the NNPD (2002-2003), which documented HIE in 1.4% of institutional deliveries [20], likely reflecting the tertiary referral nature of the study center. International comparisons also demonstrate variability:

Simiyu et al. [21] reported HIE in 10.7% of deliveries at Kilimanjaro Christian Medical Centre (KCMC) in Tanzania, while Padayachee et al. [22] in South Africa reported rates of 3.6 per 1,000 live births. Differences in prevalence may result from inclusion criteria, gestational age, and high-risk referrals. Maternal and perinatal risk factors were significant predictors of HIE in the study population. Cesarean delivery for fetal distress was more frequent in the HIE group (75%) compared with the non-HIE group (46%), and lack of regular antenatal care was observed in 63% versus 42%, respectively. Meconium-stained liquor was also notably higher among neonates with HIE (60%) than among those without HIE (22.2%). These findings align with previously published literature. Shankaran et al. reported that severe intrapartum compromise, reflected by low Apgar scores and the requirement for advanced resuscitation, strongly predicts the severity of HIE and influences long-term neurodevelopmental outcomes [23]. Ross and Gala further demonstrated that significant intrapartum hypoxia, as indicated by worsening umbilical artery base excess, is closely associated with the development of HIE and adverse neonatal outcomes [24]. Similarly, Shrestha et al. identified meconium-stained amniotic fluid, fetal distress, and inadequate antenatal care as important perinatal contributors to birth asphyxia [25]. Consistent with these observations, the present study found significantly lower mean five-minute Apgar scores in neonates with HIE (4.95 ± 1 SD) compared with non-HIE neonates (6.29 ± 1 SD), underscoring the influence of intrapartum compromise on neonatal neurological outcomes. The intensity of resuscitation correlated with HIE severity. Among the 164 neonates requiring advanced resuscitation, 21.3% received PPV for 30 seconds, 25% for one minute, and 49.3% for > one minute, requiring intubation. Chest compressions and drug administration were required in 1.82% and 1.82%, respectively. Stage I HIE occurred predominantly in neonates requiring shorter resuscitation, whereas Stage III HIE was associated with prolonged PPV, intubation, chest compressions, or drug use (p < 0.05). Mortality was highest in Stage III HIE (79.4%). This is consistent with the findings of Ju et al., who demonstrated that severe hypoxic-ischemic injury leads to extensive neuronal apoptosis, contributing to poor neurological outcomes and higher mortality [26]. Similarly, Khreisat and Habahbeh reported that severe intrapartum asphyxia and related perinatal risk factors markedly increase the likelihood of adverse outcomes, including death, in affected neonates [27].

MOD was present in all neonates with HIE. The most commonly affected organ systems included metabolic (81.1%), renal (55.5%), respiratory (51.2%), hematological (42.7%), CNS (40.2%), gastrointestinal (32.9%), and cardiovascular (31.1%) systems. Cardiovascular dysfunction contributed to the highest mortality (54.9%), followed by gastrointestinal (40.7%), hematologic (35.7%), and renal (35.1%) involvement. These findings align with previous observations. Shankaran et al. reported that severe HIE is frequently accompanied by dysfunction in multiple organ systems, particularly cardiovascular and renal compromise, which are strongly associated with poor survival outcomes [23]. Wheeler et al. described MOD as a progressive, multisystem failure resulting from severe hypoxic-ischemic injury, emphasizing that the number of organs involved directly correlates with disease severity and mortality risk [28]. Similarly, Tekin highlighted that post-resuscitative complications in asphyxiated neonates commonly involve metabolic, renal, and cardiovascular systems, underscoring their contribution to adverse outcomes [29]. In the present study, two-organ dysfunction was most common (49%), followed by single-organ involvement (38%), reflecting the cumulative burden and prognostic impact of multi-organ compromise in HIE.

The relationship between HIE stage and MOD severity was evident: Stage III HIE neonates exhibited the highest incidence of MOD, followed by Stage II and Stage I. Moderate to severe involvement of organ systems was predictive of at least moderate HIE, while mild HIE was associated with minimal MOD and faster recovery. These findings align with the observations of Alsina et al. [15] and Hankins et al. [30], indicating that early evaluation of organ dysfunction is critical for prognostication.

The renal system was affected in 55.4% of neonates, as assessed by urine output and serum creatinine levels. Cardiovascular dysfunction occurred in 31.1%, with arrhythmias seen in 5.49%, while the majority (94.51%) maintained normal heart rates. Hematological involvement was present in 42.68%, including disseminated intravascular coagulation (DIC) in 4.27%. Respiratory support requirements varied: 48.78% required no support, 18.9% required non-invasive or short-duration mechanical ventilation, and 32.32% required prolonged ventilatory assistance. These findings align with previous literature. Mohammed et al. reported similar multi-organ involvement in neonates with HIE, particularly renal and hematologic dysfunction, underscoring their role in disease severity and outcomes [31]. Chauhan et al. also noted that acute birth asphyxia severe enough to cause neonatal encephalopathy commonly results in injury across multiple organ systems, including renal, cardiovascular, and respiratory systems [32].

The findings confirm that MOD is strongly associated with HIE severity, and early assessment of organ system involvement provides prognostic information. Neonates with severe HIE are likely to present with significant dysfunction across multiple organs within hours of birth, whereas moderate HIE exhibits a broader spectrum of MOD severity. Absence of MOD should prompt reconsideration of HIE as the primary etiology of encephalopathy.

Limitations include the single-center design and lack of long-term neurodevelopmental follow-up. Lack of advanced neuroimaging or electroencephalogram correlation. Potential confounding factors included hyperbilirubinemia, sepsis, or late preterm physiology. Comparisons with retrospective studies [15] and multicenter data [20, 23] are limited by differences in methodology and patient populations.

## Conclusions

HIE occurs in a substantial proportion of late preterm and term neonates requiring resuscitation beyond the initial steps at birth, with Stage III being the most prevalent. MOD is highly common in these neonates, most frequently involving the metabolic, renal, respiratory, hematologic, and cardiovascular systems. Increasing HIE stage is strongly associated with a greater extent of organ involvement, and prolonged or intensive resuscitation is linked to higher rates of MOD. Mortality was significantly higher among neonates with severe HIE and those with involvement of multiple organ systems. Early recognition of MOD provides important prognostic insight, as neonates with moderate-to-severe involvement across multiple systems are more likely to experience adverse outcomes, including death. These findings highlight the importance of vigilant monitoring and timely intervention in neonates with perinatal asphyxia to improve survival and reduce morbidity.
